# Feasibility of Internet-Based Health-Related Quality of Life Data Collection in a Large Patient Cohort

**DOI:** 10.2196/jmir.1214

**Published:** 2010-08-19

**Authors:** Sacha Bhinder, Noori Chowdhury, John Granton, Murray Krahn, D Elizabeth Tullis, Thomas K Waddell, Lianne G Singer

**Affiliations:** ^4^Division of Thoracic SurgeryDepartment of SurgeryUniversity of TorontoTorontoCanada; ^3^Division of RespirologyDepartment of MedicineUniversity Health NetworkTorontoCanada; ^2^Toronto General Research InstituteUniversity Health NetworkTorontoCanada; ^1^Faculty of MedicineUniversity of TorontoTorontoCanada

**Keywords:** Quality of life, cohort studies, Internet, feasibility studies, lung diseases

## Abstract

**Background:**

Patient registries are commonly used to track survival and medical outcomes in large cohorts. However, large-scale collection of health-related quality of life (HRQOL) data is more challenging because such data must be collected directly from patients. Internet-based HRQOL questionnaires are a potential solution, allowing home data collection with immediate storage in a central database.

**Objectives:**

Our objectives were to investigate the sociodemographic predictors of Internet use and willingness to convey HRQOL information over the Internet in a Canadian tertiary care patient population and to determine whether Internet use patterns of tertiary care patients differ from those of the general Canadian population. Additionally, we sought to identify the success of home completion of Internet-based HRQOL questionnaires, as well as factors hindering home completion.

**Methods:**

We surveyed 644 patients at the Toronto General and St. Michael’s Hospitals from November 2003 through July 2006 within a prospective, longitudinal cohort study of HRQOL in patients with lung disease or lung transplants. Using multiple logistic regression, we assessed patient age, gender, rurality, marital status, and employment or education status as potential sociodemographic predictors of having an Internet-accessible home computer, using email at least weekly, and willingness to complete a quality of life questionnaire over the Internet. Patients electing to complete questionnaires over the Internet were followed from September 2005 through March 2008 to assess completion of HRQOL questionnaires from home, identify barriers for noncompletion, and determine sociodemographic predictors for home completion.

**Results:**

Of the 644 patients, the median age was 51 years, with a similar number of males and females. Most were urban Ontario residents, were unemployed, and were married or in a common-law relationship. Having an Internet-accessible home computer was reported by 79.7% (513/644) of patients and use of email at least weekly by 66.5% (414/623) of patients. A majority of patients (57.1% 368/644) were willing to complete HRQOL questionnaires over the Internet via an emailed link. Of the participating 644 patients, 368 elected to complete future questionnaires from home and, as part of a gradual roll-out of the home HRQOL questionnaire, 211 were sent emails inviting them to do so. Of the invited patients, 78% (165/211) completed at least one questionnaire from home. The most common reason for noncompletion was a lack of or an inability to find time to complete the questionnaire. No statistically significant sociodemographic predictors of Internet use were associated with completion or noncompletion of questionnaires from home.

**Conclusions:**

Home, Internet-based HRQOL assessment is feasible in tertiary care patient populations with a high predicted rate of Internet usage based on sociodemographic parameters. A large minority of patients were unwilling or unable to take part in home HRQOL assessments indicating that alternative methods of data collection are still required. However, the majority of patients electing to complete home HRQOL assessments went on to do so over the Internet.

## Introduction

Disease registries and multicenter cohort studies are widely used to capture large amounts of observational data regarding epidemiology, treatment, and outcomes of common and rare disorders [1]. One example is the registry of the International Society of Heart and Lung Transplantation (ISHLT), which tracks a wide variety of procedural and outcome data, including survival and complication rates, from transplant programs around the world [2].

Registries and multicenter observational studies typically focus on survival and other easily classifiable medical outcomes. While health-related quality of life (HRQOL) is widely recognized as an important outcome [1,3] large-scale collection of HRQOL data has lagged behind. Health-related quality of life can be broadly defined as “an individual's satisfaction or happiness with life in domains he or she considers important…insofar as they affect or are affected by factors that are generally considered to fall under the purview of health care providers, or that are likely to be the target of a health care intervention” [4].

Lung transplantation is one clinical area where HRQOL data are needed to improve clinical decision-making [5]. Current clinical guidelines recommend lung transplantation based on expected improvement in survival because the factors that impact upon HRQOL in advanced lung disease before and after transplantation are poorly characterized [6]. However, lung transplantation may greatly improve HRQOL even for conditions where the survival benefit is unclear, and the risks of surgery and immunosuppressive drugs introduce important trade-offs that are difficult to understand on the basis of survival considerations alone.

Unlike “hard” medical outcomes, HRQOL data must be collected directly from patients, scored, and entered into a centralized database. This requires time, money, and access to patients, all of which may be limited for centers voluntarily reporting health outcome data. The Internet provides an ideal method of obtaining HRQOL data from multiple home and clinic sites following which data can be automatically saved and scored in a central database. Electronic HRQOL instruments have several additional advantages including the ability to automatically prompt subjects to correct missing or invalid responses, skip irrelevant items, and track the time, date, and duration of each assessment [7]. However, reliable home follow-up is essential for optimal usage of Internet technology, and stated willingness to transmit information does not always translate into action. In studies where prior Internet survey participants were invited by email to participate in an Internet survey, the participation rate was as low as 30%, and the rate of invalid email addresses was 23%, illustrating several potential obstacles [8].

Internet use for health purposes is a global phenomenon [9]. In a recent cross-sectional study of individuals in the metropolitan Paris area, those having Internet access as well as a medical condition were more likely to use the Internet to search for health information [10].

While predictors of Internet use have been identified for the general population [9], tertiary care clinic patients may have different rates and predictors of Internet usage. On one hand, access to highly specialized care may indicate a savvy health care consumer with above-average computer usage; on the other hand, the deleterious effects of chronic illness on education, employment, and income may limit access to home computers and the Internet. A recent study by Wangberg et al showed that individuals with higher reported levels of subjective health status were more likely to use the Internet to seek health information [11]. In a hospital care setting among new patients in a teaching rheumatology clinic, 62.5% reported having researched their symptoms and condition on the Internet prior to their first visit [12].

With close to 73% of the Canadian adult population accessing the Internet and 59% of adult home users using the Internet for health or medical information, the Internet is rapidly becoming a primary source of medical information for Canadians [9]. Additionally, for patients with chronic conditions, Internet-based tools for patient communication of symptom and disease information have been shown to be feasible models for patient-physician communication [13,14].

However, broad use of the Internet for direct communication between health care providers and patients is limited. For patients to communicate HRQOL information over the Internet, they require secure Internet access and must be willing to transmit potentially sensitive information over the Internet.

Our research aims were as follows: (1) to characterize Internet access and usage patterns of tertiary care patients; (2) to determine whether Internet access and usage in tertiary care patients differ from those of the general Canadian population; (3) to quantify the willingness of tertiary care patients to communicate HRQOL information over the Internet; (4) to define sociodemographic and health-related predictors of both Internet use and willingness to communicate HRQOL information over the Internet; and (5) in a subset of patients who indicated willingness to communicate information over the Internet, to assess actual completion rates when patients were invited to submit information from home.

## Methods

This study was a part of an ongoing, prospective, longitudinal cohort study of HRQOL using an Internet-based questionnaire (Multimedia Appendix 1). The study was approved by each institution’s research ethics board, and all subjects provided written informed consent.

### Subjects

Patients attending selected Toronto General Hospital and St. Michael’s Hospital outpatient clinics were enrolled as study subjects from November 2003 through July 2006. The study population consisted of adult subjects who had been diagnosed with one of the four most common indications for lung transplantation (cystic fibrosis (CF), chronic obstructive pulmonary disease (COPD), interstitial lung disease (ILD), and pulmonary arterial hypertension; a small adult population of subjects with other indications for lung transplantation; and a sixth group of subjects who had received a lung transplant. All posttransplant patients were eligible for the study while other patients were included if they were potential future transplant patients (ie, age ≤ 75, clinically significant lung disease with prespecified criteria for each disease, nonsmokers, and no other significant comorbidities which would preclude transplantation). We attempted to enroll a consecutive sample of stable patients presenting to clinic. Ability to read and understand English was a prerequisite. In total, six patients were not included because of an inability to understand English.

### Study Design

Participating patients in each group completed an Internet-based questionnaire that assessed HRQOL and computer and Internet use habits. The final question asked subjects whether they would prefer to complete the questionnaire in the future over the Internet from their own home using their home computer. Subjects who indicated they preferred this option were asked to provide their email address. Following the initial questionnaire, subjects completed questionnaires at least annually either at clinic visits or at home over the Internet.

Questionnaires were housed on a secure, encrypted website. The website was designed for use in a Windows environment using MS Internet Explorer version 5.5 or higher. Patients were informed that the website was not accessible through Netscape Navigator or Mozilla Firefox. Website security involved 128-bit public key encryption, and access was restricted to registered users with a valid ID number and password. Subjects consenting to participate in the study were supplied with a unique 6 digit ID number corresponding to their hospital ID number and asked to generate their own unique password. Account creation was verified during the first clinic visit when subjects underwent an initial HRQOL assessment. Computer operating system and/or Internet browser type were not factors in patient recruitment.

Patients electing to complete future questionnaires at home over the Internet were contacted by email one week before follow-up clinic appointments and were provided with a Web address to access the questionnaire. In the event of unsuccessful email transmission due to an incorrect email account, the patient was contacted by phone to provide an updated address. This phase of the study took place from September 2005 through March 2008. For logistical reasons the second phase of the study was restricted to clinic patients from the Toronto General Hospital and did not include patients from the other participating clinics. During this second phase of the study, then, 211 patients who had said they were willing to complete the questionnaire from home were contacted and asked to complete the questionnaire. Patients declining home completion completed the questionnaire during their clinic appointment using a hospital computer.

In this paper, we report data from the 644 participating patients’ initial assessments, which took place in a supervised clinic environment, as well as follow up assessments for a subset of 211 patients (described above) who requested future assessments over the Internet and were then asked to complete these assessments.

The 211 patients were invited to complete a questionnaire from home prior to each transplant clinic visit following the first clinic visit during which they were recruited for the study. Depending on the severity of lung disease pretransplant or time since transplant, the interval between follow-up assessments ranged from 3 months to one year. Nonresponders were approached during clinic visits and requested to provide a reason for not responding to the initial emailed survey request.

### Measurements

#### The HRQOL assessment

Components of the online HRQOL assessment included the Standard Gamble utility for current health, a population preference-weighted utility measure (EQ-5D), a general health survey (SF-36), a disease-specific health index (St George’s Respiratory Questionnaire, SGRQ), and a visual analog scale. Completion of the entire HRQOL assessment in the clinic environment required an average of 26 minutes. The HRQOL assessment could only be completed in one session and could not be saved and finished at a later date.

#### Outcome Variables

Computer use questions assessed past computer use, possession of a home computer with or without Internet access, Internet connection speed (high-speed vs dial-up), and the frequency of computer, email, and Internet use. We chose home Internet access, checking email at least weekly, and stated willingness to do home HRQOL assessments as the primary outcome measures for the initial in-clinic assessment as all of these were necessary if Internet-based HRQOL assessment from home was feasible. We revised the computer use questionnaire shortly after the study’s inception, so that some patients did not answer all the computer use questions. We examined home HRQOL completion rate and reasons for noncompletion in patients who were invited to do home assessments.

#### Predictor Variables

Sociodemographic parameters collected during the assessment included age, gender, province of primary residence, employment or school status, and marital status. Primary lung disease, transplant status, and HRQOL were also considered as predictor values. Rurality and median household income were determined based on patient’s permanent home address postal code retrieved from medical records.

For age data, the median value was used to define the boundary between dichotomized categories. Frequencies of computer use, email use, and Internet searching/browsing data were dichotomized into the following categories: at least once a week and less than once a week. Rurality was determined through comparison of patient postal codes and Canada Post urban and rural forward sortation area (FSA) postal codes. Median household income was inferred using Statistics Canada median household income data by FSA region [15]. As median household income was inferred from FSA codes and not directly collected from patients, it was used for descriptive purposes only. Median household income was categorized into quartiles as found in the National Population Health Survey [16]. The four quartiles were: less than Can $30,000; Can $30,001 to Can $60,000; Can $60,001 to Can $80,000; and greater than Can $80,000. Characteristics of Internet use among the general Canadian population were obtained from the 2007 Canadian Internet Use Survey (CIUS) [9].

### Analysis

We used two-sided one-sample test for binomial proportions to compare our data with the proportions provided by Canadian Internet Use Survey (CIUS) dataset representing the Canadian population's usage of the Internet. Two sample *t* tests were performed to compare baseline HRQOL between subjects willing and not willing to complete future HRQOL assessments over the Internet. To examine associations between health-related predictors (lung disease and transplant status) of Internet use and willingness to communicate HRQOL information over the Internet, we assessed bivariate associations using Chi-square tests with Yates continuity correction. Binary logistic regression was used to assess the independent effects of six sociodemographic predictor variables on each of the four outcomes: home Internet access, regular email usage, stated willingness to complete future HRQOL questionnaires over the Internet, and actual completion of at least one follow-up HRQOL assessment over the Internet. For each outcome, the fitted model included all the predictor variables. We did not test for interactions, nor did we adjust for multiple comparisons.

Statistical analyses were performed using the SAS System version 9.1 for MS Windows (SAS Institute, Inc, Cary, NC, USA) and *P* < .05 was taken to indicate statistical significance.

**Figure 1:**
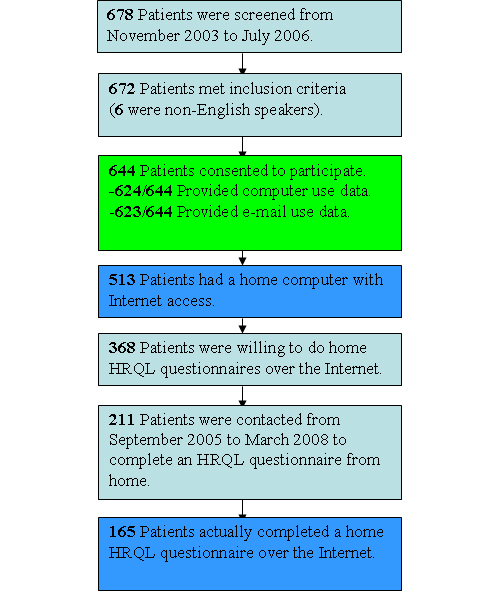
Patient flow through our study

## Results

Out of 672 patients approached to participate in the study, 644 consented and completed the initial assessment. Among nonconsenting patients, reasons for nonparticipation were: not interested in research studies (14 patients); already enrolled in other studies (1 patient); not feeling well enough to participate (1 patient); had been through enough tests (2 patients); or not emotionally prepared to participate (2 patients). An additional 8 patients did not provide a reason for not participating. Data for email use was collected from 623 patients out of a total of 644 patients who completed the survey. Data for computer use as well as Internet searching/browsing was collected from 624 patients, and data for the speed of home Internet connections (high-speed versus dial-up) was collected from 232 patients. A subset of the study cohort was contacted later to complete the assessment from home. A diagram of patient flow through our study is shown in Figure 1.

Table 1 highlights the sociodemographic characteristics for our entire consenting patient cohort, as well as for subgroups categorized by pretransplant pulmonary disease and posttransplant status. The majority of our patients were between 35 and 64 years of age. Most were urban, Ontario residents, were unemployed or not in school, and were either married or involved in common-law relationships.

**Table 1:** Sociodemographic characteristics of our patient cohort

			PreTransplant (n = 364)					Post Transplant
		Whole Cohort	COPD	ILD	Pulmonary Hypertension	CF	Other	
		n (% )	n (% )	n (% )	n (% )	n (% )	n (% )	n (% )
		n = 644	n = 76	n = 66	n = 90	n = 109	n = 23	n = 280
**Age (years)**								
	18-34	121 (18.8)	2 (2.6)	0 (0)	6 (6.7)	65 (59.6)	3 (13.0)	45 (16.1)
	35-54	246 (38.2)	20 (26.3)	20 (30.3)	50 (55.6)	41 (37.6)	12 (52.2)	103 (36.8)
	55-64	179 (27.8)	35 (46.1)	29 (43.9)	22 (24.4)	3 (2.8)	6 (26.1)	83 (29.6)
	65 and over	98 (15.2)	19 (25.0)	17 (25.8)	12 (13.3)	0 (0)	2 (8.7)	49 (17.5)
**Gender**								
	Male	312 (48.4)	37 (48.7)	37 (56.1)	26 (28.9)	62 (56.9)	10 (43.5)	140 (50.0)
	Female	332 (51.6)	39 (51.3)	29 (43.9)	64 (71.1)	47 (43.1)	13 (56.5)	140 (50.0)
**Rurality**								
	Urban	524 (81.4)	55 (72.4)	51 (77.3)	76 (84.4)	94 (86.2)	19 (82.6)	229 (81.8)
	Rural	120 (18.6)	21 (27.6)	15 (22.7)	14 (15.6)	15 (13.8)	4 (17.4)	51 (18.2)
**Employment/school**								
	Full-time	146 (22.7)	11 (14.7)	13 (19.7)	24 (26.9)	53 (48.6)	1 (4.3)	42 (15.0)
	Part-time	56 (8.7)	7 (9.3)	2 (3.0)	3 (3.4)	10 (9.2)	2 (8.7)	32 (11.5)
	Unemployed	442 (68.6)	57 (76.0)	51 (77.3)	62 (69.7)	46 (42.2)	20 (87.0)	205 (73.5)
**Marital status**								
	Married/common-Law	420 (65.2)	48 (63.2)	51 (77.3)	56 (62.2)	52 (47.7)	14 (61.0)	197 (70.9)
	Divorced/separated	74 (11.5)	17 (22.4)	7 (10.6)	15 (16.7)	6 (5.5)	1 (4.3)	25 (9.0)
	Single	134 (20.8)	8 (10.5)	5 (7.6)	16 (17.8)	50 (45.9)	3 (13.0)	50 (18.0)
	Widowed	16 (2.5)	3 (3.9)	3 (4.5)	3 (3.3)	1 (0.9)	5 (21.7)	6 (2.1)
**Province of residence**								
	Ontario	577 (89.6)	68 (89.5)	56 (84.9)	85 (94.4)	105 (96.3)	18 (78.3)	245 (87.5)
	Atlantic Canada	62 (9.6)	7 (9.2)	10 (15.1)	4 (4.4)	4 (3.7)	4 (17.4)	33 (11.8)
	Other	5 (0.8)	1 (1.3)	0 (0)	1 (1.1)	0 (0)	1 (4.3)	2 (0.7)
**Estimated median household income**								
	Lowest quartile	9 (1.4)	0 (0)	0 (0)	0 (0)	6 (5.5)	0 (0)	3 (1.1)
	Second quartile	425 (65.9)	57 (75.0)	41 (62.1)	55 (61.1)	69 (63.3)	16 (69.6)	186 (66.4)
	Third quartile	193 (30.1)	16 (21.1)	17 (25.8)	23 (25.6)	25 (22.9)	6 (26.1)	60 (21.4)
	Highest quartile	17 (2.6)	3 (3.9)	8 (12.1)	12 (13.3)	9 (8.3)	1 (4.3)	31 (11.1)

Table 2 details the characteristics of computer and Internet use among participating patients. In total, 79.7% (513/644) of patients had a home computer with Internet access. Data for the speed of home Internet connections (high-speed versus dial-up) was collected from 232 patients. Among these patients, 187 or 80.6% connected to the Internet through a high-speed connection. Of 623 patients who responded to the question, 66.5% (414/623) were accessing email at least weekly. Of the total 644 participating patients, 57% (368/644) were willing to complete HRQOL questionnaires over the Internet, indicating that a sizable minority were not prepared to do this.

**Table 2:** Computer use questionnaire and responses in 644 subjects

		n (%)
**Have you used a computer before today?**		
	Yes	558 (86.6)
	No	86 (13.4)
**Do you have a computer at your current residence?**		
	Yes	544 (84.5)
	No	100 (15.5)
**If yes, do you have Internet access? (n = 544)**		
	Yes	513 (94.3)
	No	31 (5.7)
**Connection speed (n = 232)**		
	High-speed	187 (80.6)
	Dial-up	45 (19.4)
**Computer use (n = 624)**		
	Never/in the past	137 (22.0)
	Monthly	35 (5.6)
	Weekly	108 (17.3)
	Daily	344 (55.1)
**Email use (n = 623)**		
	Never/in the past	189 (30.3)
	Monthly	20 (3.2)
	Weekly	107 (17.2)
	Daily	307 (49.3)
**Internet searching/browsing (n = 624)**		
	Never/in the past	187 (30.0)
	Monthly	44 (7.1)
	Weekly	144 (23.0)
	Daily	249 (39.9)
**In the future, would you prefer to complete from home? (n = 644)**		
	Yes	368 (57.1)
	No	276 (42.9)

**Table 3:** Comparison of Internet use habits among our tertiary care patient cohort and the general Canadian population

Computer Use Parameter:	Prevalence of Computer Access and Use		*P*
	Study Population n (%)	General (Canadian) Population in 2007 (%)^a^	
Internet access among urban residents	423/443 (95.5)	76.0	< .001
Internet access among rural residents	90/101 (89.1)	65.0	< .001
High-speed Internet access	187/232 (80.6)	88.0	< .001
Regular Internet use	437/624 (70.0)	73.0	.09
Daily Internet use	249/624 (39.9)	68.0	< .001

^a^Statistics Canada. The Daily, Canadian Internet Use Survey. 2008.

Table 3 presents a comparison of the Internet use habits of our cohort of tertiary care patients to the general Canadian population as reported in the Canadian Internet Use Survey [9]. Of patients in our cohort, 70% (437/624) were regular Internet users compared with 73% of Canadian adult population. However, 68% of Canadians used the Internet on a daily basis while 40% of our patients (249/624) were daily Internet users. Our urban and rural patients were more likely to be Internet users compared with those in the general Canadian population. High-speed Internet access was available for 81% of our patients (187/232), which was lower than the 88% reported for the Canadian population.

No statistically significant differences in computer use characteristics or willingness to complete the questionnaire from home were observed between pretransplant and posttransplant patients, or between patients with different lung diseases. According to transplant status, 56% of pretransplant patients (204/364) and 59.3% (166/280) of posttransplant patients were willing to complete future questionnaires from home (*P* = .40). There was no difference in any of the assessed quality of life measures between subjects willing and not willing to complete HRQOL assessments from home (*P* < .10 for all comparisons, data not shown).

Table 4 presents the multiple logistic regression for predictors of computer and Internet use. Access to an Internet-accessible home computer, use of email at least weekly, and stated willingness to complete the HRQOL questionnaire from home were all associated with age younger than 51 years and employment or school enrollment. Patients younger than 51 years of age were twice as likely as their older peers to have an Internet accessible home computer and to use email at least weekly. Patients employed or in school were over three times as likely to have an Internet accessible home computer and use email at least weekly. No significant variables were identified to differentiate the characteristics of patients who completed one or more HRQOL questionnaires from their home computers versus those who did not.

**Table:** Binary logistic regression analysis of factors associated with computer and Internet use

	Internet Access for Computer at Current Residence (n = 644)			Email Use (n = 623)			Willing to Complete Questionnaire From Home Over the Internet (n = 644)			Actual Completion of One or More Questionnaires From Home Over the Internet (n = 211)		
	Odds Ratio	95% CI	*P*	Odds Ratio	95% CI	*P*	Odds Ratio	95% CI	*P*	Odds Ratio	95% CI	*P*
Age < 51 years	1.99	1.17–3.38	.01	2.02	1.39–2.93	.001	1.56	1.11–2.19	< .01	1.01	0.99–1.03	.61
Male gender	1.07	0.66–1.75	.77	0.99	0.69–1.42	.95	1.09	0.79–1.50	.61	0.95	0.48–1.86	.95
Urban residence	1.00	0.55–1.85	.98	1.04	0.66–1.64	.86	1.37	0.91–2.06	.13	0.69	0.28–1.70	.42
Employed or in school	3.24	1.60–6.56	.001	3.71	2.35–5.88	< .001	1.48	1.02–2.13	.04	1.38	0.64–3.01	.31
Married/ common-law	1.38	0.82–2.32	.23	0.70	0.47–1.04	.08	0.87	0.62–1.24	.45	0.80	0.39–1.69	.35
Ontario resident	1.50	0.75–3.03	.25	0.92	0.52–1.64	.79	1.00	0.59–1.69	.99	1.28	0.46–3.55	.60

For the 368 patients that preferred to complete future questionnaires from home, 211 patients received emails inviting them to do so during the study period, and 78% (165/211) of these patients went on to complete at least one questionnaire from home. Of the 211 patients invited to complete questionnaires from home, 27 patients (13%) were contacted by phone to update their email address following unsuccessful email transmission. An additional 46 patients (21.8%) received one or more email invitations but did not complete a questionnaire from home. For these patients, reasons for noncompletion were collected during clinic visits and are depicted in Table 5. The most common reason for noncompletion was a lack of time to complete the questionnaire; 17 of the 46 patients (37%) gave this reason while 15% (7/46) did not provide a reason for noncompletion. Among the 211 patients who were invited to complete questionnaires from home, there were no significant sociodemographic predictors of actual completion.

**Table 5:** Reasons for noncompletion of home HRQOL questionnaires over the Internet (n = 46)

Reasons for Noncompletion	n (%)
Did not find time	17 (37)
Incompatible hardware/software	6 (13)
Computer was non-functional or under repair	5 (11)
Infrequently checked email	5 (11)
Changed email address	3 (7)
Email directed to “junk” folder	2 (4)
Patient withdrew from study	1 (2)
Patient did not provide a reason	7 (15)

## Discussion

In this study, we surveyed participants enrolled in a longitudinal cohort study of HRQOL regarding home Internet access and usage, self-reported willingness to do Internet-based HRQOL assessments, and actual completion rates for these assessments. We found a majority of patients have the necessary equipment and are willing to communicate HRQOL information over the Internet. However, the success of Internet-based HRQOL data collection will depend upon the characteristics of the patient cohort under study. Similar to the general population [9], young, single urban dwellers who are working or in school are much more likely to have Internet access and be willing to participate than subjects without these characteristics.

Within our study cohort of tertiary care patients, for example, the group of cystic fibrosis patients is fairly well described by these parameters (as can be appreciated by the characterization of this cohort in Table 1) and might be an excellent candidate population for longitudinal home HRQOL monitoring over the Internet. On the other hand, older patients such as the group with COPD or ILD might be less suited to HRQOL data collection exclusively from home, and might require supplemental strategies such as data collection in clinic, by telephone, or via mailed paper questionnaires. While not directly assessed in our study, the impact of the age of patients with specific diseases or the resulting functional and cognitive impact of these diseases on the abilities of patients should be considered when designing Internet-based data collection strategies.

Most patients agreeing to complete questionnaires from home went on to do so. For the minority of patients that remained noncompleters from home, time constraints and use of an incompatible Web browser (ie, Firefox and Netscape) or operating system (ie, Macintosh) were largely responsible for preventing home completion. Infrequent access to email, incorrect address, or spam filters accounted for a small percentage for reasons of noncompletion. No sociodemographic factors were statistically significant in predicting home completion.

Compared with the Canadian population, our patients were more likely to have Internet-accessible computers in both urban and rural residences. However, frequency and regularity of Internet use among our patients was statistically less than that of the Canadian population. Infrequent Internet use may reflect the characteristics of our cohort of older, unemployed, or retired individuals that have lower occupational need for daily Internet and email use. However, similar prevalence of Internet access and high-speed connections suggests that population-based estimates of Internet usage may be useful for planning Internet-based studies in tertiary care populations.

With respect to our HRQOL instrument, the questionnaire required completion in one session and could not be saved and returned to at another time. This design limitation may have affected stated patient willingness to complete it over the Internet as well as the lack of time cited by some patients who failed to do home assessments. The questionnaire also would not run in a Macintosh operating system or on a mobile phone browser, and this may have accounted for some subjects’ unwillingness or inability to do home assessments. Our results suggest several strategies that may be helpful in improving response rates, including designing questionnaires to run on multiple operating systems and browsers, providing ample time for survey completion including the capability to save and return to partially completed questionnaires, and sending email reminders after the initial invitation.

Internet-based HRQOL assessment will become increasingly important as disease registries and multicenter cohort studies move to add HRQOL to the other outcomes data they collect [17]. HRQOL is widely recognized as an essential outcome measure even for “lifesaving” interventions such as cancer care or organ transplantation [18-20].

Even for patients without home Internet access, collection of HRQOL data in clinic through an Internet site offers advantages over traditional, paper HRQOL forms. Electronic HRQOL instruments include the ability to automatically prompt subjects to correct missing or invalid responses, to skip irrelevant items, to track the time, date and duration of each assessment, and to automatically save and score the HRQOL measures in a central database [7]. Several studies have favorably compared paper and electronic versions of HRQOL instruments, including the SF-36 [21] and a visual analog scale for global health [22]. These studies indicate that electronic HRQOL instruments are valid, reliable, are completed more quickly and with fewer errors, and are preferred by the majority of patients.

While prior studies have examined the feasibility of Internet-based HRQOL assessment, some have focused on HRQOL assessment in the clinic setting as a tool for clinicians to better understand patients’ health. For example, Gutteling et al deployed a voluntary Internet-based HRQOL assessment in a single academic hepatology clinic [23], while Rogausch et al demonstrated the feasibility and acceptance of electronic HRQOL instruments (including a subgroup of patients with chronic lung disease) in a multicenter general practice cohort [24]. Our study confirms that clinic-based HRQOL is feasible and acceptable to patients in a tertiary care clinic setting. While these prior studies relied on busy clinic staff to explain the questionnaire and invite patients to participate, with variable results, our study employed dedicated research assistants for this purpose. This explains our high participation rate and reflects a “best possible” scenario that would not easily be replicated as part of routine clinical care.

However, perhaps the most powerful argument for Internet-based HRQOL instruments is that the Internet can bring data collection into patients’ homes anywhere in the world. Previous studies of Internet-based HRQOL assessment have included samples of members of the general public [25] or patients who self-identify as having a particular clinical condition and who are recruited through a patient website [26,27]. While each of these study designs provides valuable information, they are limited by the inability to determine whether participants in fact have the disease of interest, and, therefore, the data collected cannot be compared with clinical HRQOL data.

Our study combined recruitment of a well-characterized cohort of patients in subspecialty clinics with ongoing Internet-based HRQOL assessment from home. This strategy would allow HRQOL data to be available to easily supplement the traditional medical data reported to disease registries and multicenter studies. Our study demonstrates the feasibility of this approach and highlights some of the current limitations. We found that the majority of tertiary care clinic patients in our Canadian cohort possessed the necessary computer equipment to communicate HRQOL information over the Internet and were willing to participate in Internet-based home HRQOL data collection. For researchers involved in Internet-based HRQOL data collection, our findings demonstrate proof of concept for large-scale data collection through an Internet-based platform. However, we also highlight some of the challenges that still exist.

Despite the high prevalence of Internet access, our study indicates that there are still barriers to home Internet-based HRQOL data collection from patients. As many as 74% of respondents to the Canadian Internet Use Survey reported that they were concerned, or very concerned, about Internet privacy [28]. This may partly explain why patients with Internet access may still decline to transmit HRQOL information from home. However, the quantitative approach we used in our study did not allow us to determine why patients chose not to transmit HRQOL data over the Internet. A qualitative study could use open-ended questions to better elucidate patients’ concerns in this regard, and would be an essential adjunct to our analysis.

Our study has some notable limitations. The cohort consisted largely of patients residing within the Greater Toronto Area, and there was an underrepresentation of rural residents, which likely reflects, to some extent, referral patterns to the participating clinics. However, Internet contact with rural patients is particularly important as these are the most difficult patients to access. Furthermore, our study measured only patient willingness to complete an HRQOL questionnaire for research purposes and should not be assumed to reflect willingness to transmit medical or other personal information over the Internet. Patients understood that Internet completion of the questionnaire was voluntary and would not affect their medical care. Patient willingness could change if the transmitted information could alter the care they receive.

One additional limitation relates to our use of an English HRQOL questionnaire. As ability to read and understand English was a prerequisite for our questionnaire, six nonEnglish speakers were excluded from our study.

Internet-based home HRQOL assessment in large patient cohorts depends on the availability of an accessible and secure Internet connection, email use that is regular enough to facilitate dialogue, a willingness to communicate HRQOL information over the Internet, and the time and technical requirements to allow such communication. Studies in which the participants are likely to be young, educated or employed, single, and urban would have a high predicted rate of response. For more heterogeneous populations, additional means of HRQOL assessment remain necessary to ensure that HRQOL outcomes data are generalizable across study cohorts.

Given the movement in clinical medicine toward therapies and interventions that generate survival benefit and improvement in quality of life, Internet-based home HRQOL data collection has the potential to allow for collection of vast quantities of data that may be applicable to better defining patient populations that may or may not benefit from therapeutic intervention. Additionally, with major clinical and surgical interventions being deployed in community hospitals and community clinics engaging in long-term follow-up, Internet-based HRQOL data collection provides for both secure data collection from distributed community sites as well as centralized data collection for analysis and interpretation. Convenient home HRQOL data collection has the potential to increase access to patients capable of participating in research and decrease the number of patients lost to follow-up. We anticipate that home Internet-based HRQOL assessment will play an increasing role in research and clinical care.

## Multimedia Appendix 1

Online questionnaire: health-related quality of life in advanced lung disease and lung transplantation

## Abbreviations

CF: cystic fibrosis
CIUS: Canadian Internet Use Survey
COPD: chronic obstructive pulmonary disease
FSA: forward sortation area
HRQOL: health-related quality of life
ILD: interstitial lung disease
ISHLT: International Society of Heart and Lung Transplantation
SGRQ: St George’s Respiratory Questionnaire

## References

[1] Kennedy Lisa, Craig Ann-Marie (2004). Global registries for measuring pharmacoeconomic and quality-of-life outcomes: focus on design and data collection, analysis and interpretation. Pharmacoeconomics.

[2] Trulock EP, Edwards LB, Taylor DO, Boucek MM, Keck BM, Hertz MI (2006). Registry of the International Society for Heart and Lung Transplantation: Twenty-third official adult lung and heart-lung transplantation report—. J Heart Lung Transplant.

[3] Gotay Carolyn C, Lipscomb Joseph, Snyder Claire F (2005). Reflections on findings of the Cancer Outcomes Measurement Working Group: moving to the next phase. J Natl Cancer Inst.

[4] (2007). American Thoracic Society.

[5] Yusen R D (2009). Lung transplantation outcomes: the importance and inadequacies of assessing survival. Am J Transplant.

[6] Orens Jonathan B, Estenne Marc, Arcasoy Selim, Conte John V, Corris Paul, Egan Jim J, Egan Thomas, Keshavjee Shaf, Knoop Christiane, Kotloff Robert, Martinez Fernando J, Nathan Steven, Palmer Scott, Patterson Alec, Singer Lianne, Snell Gregory, Studer Sean, Vachiery J L, Glanville Allan R, Pulmonary Scientific Council of the International Society for Heart and Lung Transplantation (2006). International guidelines for the selection of lung transplant candidates: 2006 update--a consensus report from the Pulmonary Scientific Council of the International Society for Heart and Lung Transplantation. J Heart Lung Transplant.

[7] Eysenbach Gunther, Wyatt Jeremy (2002). Using the Internet for surveys and health research. J Med Internet Res.

[8] Ekman Alexandra, Dickman Paul W, Klint Asa, Weiderpass Elisabete, Litton Jan-Eric (2006). Feasibility of using web-based questionnaires in large population-based epidemiological studies. Eur J Epidemiol.

[9] (2008). The Daily, Statistics Canada.

[10] Renahy Emilie, Parizot Isabelle, Chauvin Pierre (2008). Health information seeking on the Internet: a double divide? Results from a representative survey in the Paris metropolitan area, France, 2005-2006. BMC Public Health.

[11] Wangberg Silje C, Andreassen Hege K, Prokosch Hans-Ulrich, Santana Silvina Maria Vagos, Sørensen Tove, Chronaki Catharine E (2008). Relations between Internet use, socio-economic status (SES), social support and subjective health. Health Promot Int.

[12] Hay M Cameron, Cadigan R Jean, Khanna Dinesh, Strathmann Cynthia, Lieber Eli, Altman Roy, McMahon Maureen, Kokhab Morris, Furst Daniel E (2008). Prepared patients: internet information seeking by new rheumatology patients. Arthritis Rheum.

[13] Castrén Johanna, Huttunen Teppo, Kunttu Kristina (2008). Users and non-users of web-based health advice service among Finnish university students - chronic conditions and self-reported health status (a cross-sectional study). BMC Med Inform Decis Mak.

[14] Wu Robert C, Delgado Diego, Costigan Jeannine, Maciver Jane, Ross Heather (2005). Pilot study of an Internet patient-physician communication tool for heart failure disease management. J Med Internet Res.

[15] Statistics Canada.

[16] Statistics Canada.

[17] Dharma-Wardene M, Au H J, Hanson J, Dupere D, Hewitt J, Feeny D (2004). Baseline FACT-G score is a predictor of survival for advanced lung cancer. Qual Life Res.

[18] Moraca Robert J, Low Donald E (2006). Outcomes and health-related quality of life after esophagectomy for high-grade dysplasia and intramucosal cancer. Arch Surg.

[19] Vermuelen Karin M, van der Bij Wim, Erasmus Michiel E, TenVergert Elisabeth M (2007). Long-term health-related quality of life after lung transplantation: different predictors for different dimensions. J Heart Lung Transplant.

[20] Gerbase Margaret W, Spiliopoulos Anastase, Rochat Thierry, Archinard Marc, Nicod Laurent P (2005). Health-related quality of life following single or bilateral lung transplantation: a 7-year comparison to functional outcome. Chest.

[21] Bliven B D, Kaufman S E, Spertus J A (2001). Electronic collection of health-related quality of life data: validity, time benefits, and patient preference. Qual Life Res.

[22] Athale Ninad, Sturley Ann, Skoczen Steven, Kavanaugh Arthur, Lenert Leslie (2004). A web-compatible instrument for measuring self-reported disease activity in arthritis. J Rheumatol.

[23] Gutteling Jolie J, Busschbach Jan J V, de Man Robert A, Darlington Anne-Sophie E (2008). Logistic feasibility of health related quality of life measurement in clinical practice: results of a prospective study in a large population of chronic liver patients. Health Qual Life Outcomes.

[24] Rogausch Anja, Sigle Jörg, Seibert Anna, Thüring Sabine, Kochen Michael M, Himmel Wolfgang (2009). Feasibility and acceptance of electronic quality of life assessment in general practice: an implementation study. Health Qual Life Outcomes.

[25] Coyne Karin S, Sexton Chris C, Kopp Zoe S, Luks Samantha, Gross Ashley, Irwin Debra, Milsom Ian, EpiLUTS Team (2009). Rationale for the study methods and design of the epidemiology of lower urinary tract symptoms (EpiLUTS) study. BJU Int.

[26] Enck Paul, Kowalski Axel, Martens Ute, Klosterhalfen Sibylle (2006). Internet-based assessment of bowel symptoms and quality of life. Eur J Gastroenterol Hepatol.

[27] Kinney William C, Benninger Michael S (2007). Assessment of quality of life among patients with sinonasal disease as determined by an Internet survey based on the Rhinosinusitis Disability Index. Ear Nose Throat J.

[28] Statistics Canada.

